# Metabolic Syndrome in School Children

**DOI:** 10.4274/Jcrpe.819

**Published:** 2013-05-30

**Authors:** Betül Battaloğlu İnanç

**Affiliations:** 1 Mardin Artuklu University Health Higher School, Mardin, Turkey

## INTRODUCTION

The problem of excessive weight and obesity is increasing world-wide. According to the World Health Organization (WHO), there are over 1.6 billion overweight and 400 million obese people in the world; in 2015, these figures are expected to reach 2.3 billion and 700 million, respectively. Ninety percent of obese adolescents are at risk of becoming obese adults; 75% of obese 12-year-olds go on to become obese adults; of 7-year-olds, 41% of obese ones become obese in adulthood; 25% of those obese in the pre-school period become obese adults; and 14% of obese infants are at risk of becoming obese adults. The presence of childhood obesity increases the risk of non-communicable diseases such as metabolic syndrome (MS), type 2 diabetes mellitus (DM), hypertension (HT), hyperlipidemia, cardiovascular disease, and certain cancers (1). In clinical studies, the prevalence of MS in childhood was found to be approximately 3-4%. The aim of the present study was to evaluate the prevalence of MS in southwest part of Turkey.

This is a descriptive study conducted in the city center of Mardin in the South-eastern region of Turkey. Out of a total of 4030 students in three schools, 3460 (1668 female, 1792 male) volunteer children in the 6-15-year age group were surveyed for the study, and 86% of the targeted participants were reached. The data were collected in April and May of 2011. Weight, height, waist circumference (WC), and hip circumference (HC) were measured in all children according to standardized procedures. Age was calculated in decimal units based on the date of the survey relative to birth date. Body mass index (BMI) (kg/m2) was calculated with reference to measured height and weight and was evaluated using the WHO normative data for age and gender. Obesity was defined as BMI value exceeding ≥95th percentile, and BMI value between 85-95th percentiles was indicative of overweight (2). Blood pressure (BP) was obtained and evaluated according to standard methods. Abdominal obesity was defined using the sex- and age-specific 90th WC percentile values (3). Baseline blood samples were collected by venipuncture in the morning (8:00 to 9:00 am) after an overnight fast (10 to 12 hours). The glucose oxidase method was used to determine blood glucose levels. Serum lipids including total cholesterol, high-density lipoprotein cholesterol (HDL cholesterol), low-density lipoprotein cholesterol (LDL cholesterol), and triglycerides were measured using an enzymatic colorimetric method. MS was defined according to the International Diabetes Federation criteria (4) which have been used in several paediatric studies. Statistical analysis was performed with the SPSS statistical software and Microsoft Office Excel programs. A p-value of less than 0.05 was considered statistically significant. Ethical approval for the study was given by Mardin Artuklu University, Mardin Educational and Health Directorate Office.

The female ratio of the study group was 48.2%. The average age of the students was 10.8±2.1 years, their mean height was 141.4±14.5 cm, their average weight was 37.6±13.24 kg, and their average BMI was 18.27±3.53 (range 11.28-42.08). According to their BMI values, the overweight frequency of the group was 9.4% (n=321). Frequency of obesity was 7.8% (n=269). The prevalence of obesity according to gender was found to be 8.8% (n=147) for female children and 6.8% (n=122) for male children and was significantly higher in girls (X^2^=7.244; p<0.01). A positive correlation was found between BMI and some other parameters, namely WC and HC, systolic and diastolic BP, and triglyceride levels (<0.000), but with no other parameters. The frequency of impaired fasting glucose (IFG) was 16.8% and of DM -0.7%. IFG rate was found to be higher in boys than in girls (X^2^=7.230; p=0.07). The abnormal waist/hip ratio values were higher in girls than in boys (X^2^=47.26; p<0.06). Hypertriglyceridemia was found in 51% of girls and in 63% of boys with MS. The MS rate was found to be 6.3%. There was no statistically significant difference between the genders (X^2^=0.579; p>0.05). Overweight and obese children had higher rates of MS than non-overweight children. In obese children, MS rate was 30.3%. The prevalence of overweight and obesity in the present study is similar to the figures in Turkey (2,5,6). 
